# Percutaneous Coil Closure of a Large Left Coronary Artery Fistula in an Asymptomatic Child

**DOI:** 10.7759/cureus.60594

**Published:** 2024-05-19

**Authors:** Asmaa Semrin, Jose Colon Cortes, Mark Vranicar, Musa Sharkawi, Pandya Khyati

**Affiliations:** 1 Pediatrics, Augusta University Medical College of Georgia, Augusta, USA; 2 Pediatric Cardiology, Augusta University Medical College of Georgia, Augusta, USA; 3 Cardiology, Children's Hospital of Georgia, Augusta, USA; 4 Cardiology, Augusta University Medical College of Georgia, Augusta, USA

**Keywords:** child, asymptomatic patient, cardiac cta, transcatheter closure, cardiac murmur, coronary artery fistula (caf)

## Abstract

Coronary artery fistulas (CAFs) are rare cardiac anomalies characterized by an abnormal connection between the coronary arteries and either a cardiac chamber or a large thoracic vessel. While the majority of CAF cases are asymptomatic, serious cardiac complications can occur, especially with moderate to large fistulas. We describe a case of a large-sized left coronary artery (LCA) fistula in an asymptomatic 11-year-old who was referred for cardiac evaluation due to a systolic murmur. An echocardiogram revealed a hemodynamically significant fistula arising from the LCA draining into the right ventricle. Diagnostic catheterization confirmed the origin and draining site of the fistula, along with aneurysmal dilation at the end of the fistula. The fistula was successfully closed percutaneously using a two-coil occlusive device, with no complication observed.

## Introduction

Coronary artery fistulas (CAFs) represent a relatively uncommon cardiac anomaly with an incidence of 0.002% within the general population [1]. In infants and children, most of the CAFs are congenital, accounting for 0.4% of all congenital cardiac abnormalities [1-3]. Additionally, CAFs can occur in isolation or be associated with other congenital cardiac abnormalities, such as a single coronary artery [4,5]. The other type of CAF is acquired, resulting from surgery, trauma, or infection, but these are rarely observed in children [2].

CAF classification is based on size into small, medium, and large fistulas. This classification is not solely determined by the actual size of the fistula but rather by its size relative to the adjacent or distal normal coronary artery branch [2,6]. While most CAFs are asymptomatic, some patients may experience arrhythmias, myocardial ischemia, or heart failure [7,8]. Fistulas originating from the left coronary artery (LCA) are more frequently observed than those originating from the right coronary artery (RCA).

From infancy to adulthood, CAFs can either regress spontaneously or lead to life-threatening complications, creating controversy over the best management approach. Treatment of CAFs varies depending on several factors, including the size and location of the fistula, age, symptoms, and the likelihood of complications in adulthood [2,6]. Surgical or transcatheter closure is the mainstay of treatment for medium to large CAFs in infants and children [2,6,9,10]. 

In our case, we highlight the significant occurrence of a large CAF in an asymptomatic 11-year-old male and its management.

## Case presentation

A previously healthy 11-year-old male was referred to our cardiology clinic after he was incidentally found to have a systolic murmur during his annual well visit. Vital signs were within normal limits for the patient’s age, and the electrocardiogram (ECG) was normal. Initial echocardiogram (ECHO) revealed a dilated LCA and left anterior descending (LAD) artery (Video 1), with a fistula originating from the mid-LAD draining into the right ventricular outflow tract (Video 2). The LCA measured 5.8 mm (Boston z-score: 5.10). Furthermore, the treadmill exercise stress test was negative for ischemia or atrioventricular conductive abnormalities on ECG.

**Video 1:** Transthoracic echocardiography. Short axis view showing dilated left coronary artery and left anterior descending artery.

**Video 2:** Transthoracic echocardiography. Modified four-chamber view showing coronary artery fistula originating from mid-left anterior descending artery draining into the right ventricular outflow tract.

**Figure 1:**
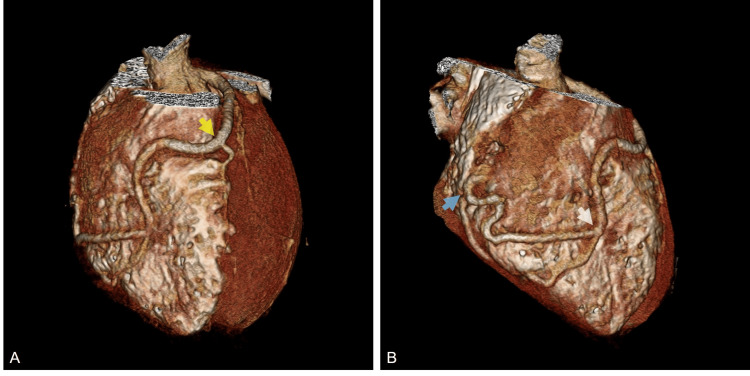
Computed tomography angiography three-dimensional images demonstrate fistula origin from the mid-left anterior descending artery (yellow arrow), which bifurcates into two branches (white arrow) and terminates near the base of the right ventricle outflow (blue arrow).

Computed tomography angiography (CTA) revealed normal origins of the coronary arteries, with ectatic LCA and proximal LAD. The mid-LAD gave rise to a large fistula coursing medially, then bifurcating into two branches terminating near the anterior base of the right ventricle, close to the atrioventricular groove (Figure 1). The mid and distal portions of the RCA were poorly visible, and the proximal RCA was of small caliber. 

Based on recommendations from the literature and discussions at the Multidisciplinary Cardiac Care Conference, the decision was made to proceed with diagnostic cardiac catheterization and device-based closure of the large CAF. The selective coronary angiography showed aneurysmal dilatation at the distal part of the fistula, which measured 7.1 mm in diameter and 8.5 mm long (Video 3). A two-coil system, consisting of an 8 mm × 40 cm Penumbra Ruby coil and a 4 mm × 20 cm Penumbra Ruby coil, was successfully inserted (Video 4). The final angiogram demonstrated a stable coil position without residual flow through the fistula and improved perfusion of the myocardium from the LCA. 

**Video 3:** Initial coronary angiogram shows a large coronary artery fistula originating from the mid-left anterior descending artery with an aneurysmal dilatation at the distal part of the fistula near its drainage into the right ventricle.

**Video 4:** Final coronary angiogram after the insertion of a two-coil system demonstrates a stable coil position without residual flow through the fistula and improved perfusion of the myocardium from the left coronary artery.

During the one-month follow-up visit, the patient was in good health, reporting no complaints or complications. Follow-up ECHO revealed normal biventricular systolic function with no residual flow through the fistula.

## Discussion

While most CAFs in children are asymptomatic, treatment is still recommended for medium to large CAFs due to a higher risk of complications in adulthood [8,9].

Previous research has shown that untreated CAFs in individuals younger than 20 years old lead to symptoms in 19% of cases, with more than 63% eventually experiencing clinical symptoms in adulthood [9]. One of the significant complications is coronary steal-related chronic myocardial ischemia, which can lead to congestive heart failure [8]. Additionally, volume overload in the cardiac chamber can also contribute to heart failure [2,8]. Although our patients' stress test did not reveal signs of ischemia, they still carry a risk of developing these complications in adulthood [2]. 

The development of aneurysms at the distal part of the fistula is a common complication, as observed in our patients, putting them at risk of rupture or thrombosis [9]. Other associated complications of CAFs include ventricular tachyarrhythmias and atrial fibrillation [8].

The treatment of CAFs depends on various factors, including the size of the fistula, symptomatology, and the likelihood of complications in adulthood [2]. ​​Moderate to large CAFs rarely close spontaneously and tend to become more hemodynamically significant as the patient ages potentially leading to serious complications such as thrombosis, myocardial infarction, or fistula rupture [2,6]. Moreover, the complexity and risks of interventions rise with the presence of comorbid conditions in adulthood. Thus, choosing to perform elective interventions on medium or large asymptomatic fistulas during childhood is an appropriate approach to prevent complications during adulthood [2,6]. The choice between transcatheter closure and surgical closure also depends on different factors, such as the type and complexity of the fistula, as well as the patient's symptoms and comorbidities [2]. Both interventions have reported excellent outcomes [10].

## Conclusions

Our case emphasizes the importance of early detection and intervention for coronary artery fistulas, particularly in pediatric patients, to mitigate the risk of future complications and ensure optimal long-term outcomes.
